# Association of Empirically Derived Dietary Patterns with Cardiovascular Risk Factors: A Comparison of PCA and RRR Methods

**DOI:** 10.1371/journal.pone.0161298

**Published:** 2016-08-22

**Authors:** Nicolas Sauvageot, Sonia Leite, Ala’a Alkerwi, Leila Sisanni, Faiez Zannad, Stranges Saverio, Anne-Françoise Donneau, Adelin Albert, Michèle Guillaume

**Affiliations:** 1 Luxembourg Institute of Health (LIH), Strassen, Luxembourg; 2 APHP, Department of Neurology and Stroke center, Bichat Hospital, INSERM LVTS-U1148, DHU FIRE, Université Paris-Diderot, Sorbonne-Paris Cité, Paris, France; 3 Hypertension Unit, Département des maladies cardiovasculaires, Centre Hospitalier Universitaire, Vandœuvre-lès-Nancy, France; 4 Ecole de Santé Publique, Université de Liège, Liège, Belgium; The University of Tokyo, JAPAN

## Abstract

**Introduction:**

Principal component analysis is used to determine dietary behaviors of a population whereas reduced rank regression is used to construct disease-related dietary patterns. This study aimed to compare both types of DP and theirs associations with cardiovascular risk factors (CVRF).

**Materiel and Methods:**

Data were derived from the cross sectional NESCAV (Nutrition, Environment and Cardiovascular Health) study, aiming to describe the cardiovascular health of the Greater region’s population (Grand duchy of Luxembourg, Wallonia (Belgium), Lorraine (France)). 2298 individuals were included for this study and dietary intake was assessed using a 134-item food frequency questionnaire.

**Results:**

We found that CVRF-related patterns also reflect eating behaviours of the population. Comparing concordant food groups between both dietary pattern methods, a diet high in fruits, oleaginous and dried fruits, vegetables, olive oil, fats rich in omega 6 and tea and low in fried foods, lean and fatty meat, processed meat, ready meal, soft drink and beer was associated with lower prevalence of CVRF. In the opposite, a pattern characterized by high intakes of fried foods, meat, offal, beer, wine and aperitifs and spirits, and low intakes of cereals, sugar and sweets and soft drinks was associated with higher prevalence of CVRF.

**Conclusion:**

In sum, we found that a “Prudent” and “Animal protein and alcohol” patterns were both associated with CVRF and behaviourally meaningful. Moreover, the relationships of those dietary patterns with lifestyle characteristics support the theory that food choices are part of a larger pattern of healthy lifestyle.

## Introduction

Many chronic pathologies such as cardio-vascular disease (CVD) constitute a major public health issue in Western Europe [1]. It has been established that common risks for CVD are high blood pressure, obesity, elevated levels of blood glucose, cholesterol and triglycerides [2]. Moreover, diet is believed to play a major role in the modulation of these cardiovascular risk factors (CVRF) [3,4].

The traditional approach of studying the relationship between diet and disease evaluates the effect of single foods. This approach however does not take into account the complexity of diet and the fact that people eat combinations of highly related foods. Interrelations between specific foods within the diet preclude to isolate the effect of a specific factor and the solution which consists of adjusting for all other foods in a multiple regression model would be flawed by the so-called multi-collinearity phenomenon [5]. In addition, the effect of one single food may be too small to be detected [6]. In response to these challenges, the use of dietary patterns (DP) that take into account how foods are consumed together has been suggested to better characterize human diet.

A number of methods have been used to derive DP. Principal component analysis (PCA) and reduced rank regression (RRR), which are data-driven exploratory methods, construct DP from the data. More specifically, PCA aims to construct uncorrelated linear combinations of food intakes that explain as much variation in food intake as possible. Therefore, PCA-derived patterns reflect dietary behaviors in the population. However, these behavior-related patterns are not necessarily predictors of the disease of interest. The reason is that explaining as much variation in food intake as possible does not mean that much variation in predictors of disease will be explained [7].

To construct disease-related dietary patterns, RRR has been suggested as an alternative approach to PCA [7]. RRR aims to construct uncorrelated linear combinations of food intakes that explain as much variation in a set of response variables as possible. In order to find CVRF-related dietary patterns, CVRF have been used as response variables [8,9]. The limitation of the RRR-derived patterns is that although they are by definition associated with response variables, they are not necessary behaviorally meaningful.

In the present study, both PCA and RRR were used to derive DP based on data from the NESCaV (Nutrition, Environment and Cardiovascular Health) survey [10], an interregional cross-sectional study of adults living in the Greater Region. The first objective was to compare behavior- and CVRF-related derived dietary patterns and to assess their relationships with common CVRF. The second objective was to use similarities between the two kinds of patterns to construct “concordant” patterns which should be both associated with CVRF and behaviorally meaningful. Associations of dietary patterns with nutrient intakes, sociodemographic and behavioral characteristics were also examined.

## Material and Methods

### Study population

Data were derived from the cross-sectional NESCAV study aimed to describe the cardiovascular health of the Greater Region’s population (Luxembourg, Wallonia in Belgium and Lorraine in France). A more detailed description of the study has been published elsewhere [10]. The protocol of the study was approved by the following institutional review boards: Comité National d’Ethique de Recherche (Grand-Duchy of Luxembourg), Comité de Protection des Personnes Est-III (Lorraine), Comité d’Ethique Hospitalo-Facultaire Universitaire de Liège (Wallonia) and Ethik-Kommission Ärztekammer des Saarlandes (Saarland) [10]. All participants provided written informed consent. The initial interregional sample was composed of 3006 individuals. For the present study, participants having a history of cardiovascular disease (n = 308), using a special diet (n = 380) or who were not fasting at time of blood collection (n = 74) were excluded. Thus, the final sample was constituted of 2298 individuals.

### Measurement of variables

#### Assessment of dietary intake

Dietary intake was assessed using a validated food frequency questionnaire (FFQ) based on 134-items [11,12]. The reported dietary intakes, expressed as frequencies and number of servings, were first converted into daily consumption. Then, daily food item intakes were converted into daily nutrients intake by using the French (SU.VI.MAX) Food Composition Database [13]. To perform a dietary pattern analysis, food items were aggregated into 45 food groups according to their similarity in the nutrient profile (S1 Table). In order to represent the composition of the diet, food groups intakes were then adjusted for energy intake using the residuals methods of Willet and Stampfer [14].

#### Cardiovascular risk factors (CVRF)

Obesity was described using the body mass index (BMI, kg/m^2^) and the waist to hip ratio (WHR). A subject with BMI≥30.0 kg/m was defined as obese [15].

Hypertension was based on systolic blood pressure (SBP, mmHg) and diastolic blood pressure (DBP, mmHg). Participants were classified as having elevated blood pressure if they reported taking anti-hypertensive medications and/or had SBP ≥ 140 mmHg and/or DBP ≥ 90 mmHg [16].

Diabetes was reflected by fasting plasma glucose (FPG, mg/dl) and glycated hemoglobin (HbA1c, %). Participants were classified as diabetics if they reported taking anti-diabetic medications and/or had FPG ≥ 126 mg/dl [17].

Finally, dyslipidemia was characterized by total cholesterol (TC, mg/dl), triglycerides (TG, mg/dl), low-density lipoprotein cholesterol (LDL, mg/dl) and high-density lipoprotein cholesterol (HDL, mg/dl). Subjects with lipid disorders were defined as having at least one of the following anomalies: TC ≥ 190mg/dl, TG ≥ 150 mg/dl, LDL-C ≥ 115 mg/dl, and HDL-C < 40 mg/dl for men and < 46 mg/dl for women, and/or taking hypo-lipid medications [18]. Details regarding the procedures of data collection and measurements were described previously [19].

#### Covariates

A self-reported questionnaire was used to collect information on socio-economic characteristics and health-related behaviors. Educational level was classified into 3 categories (No diploma, secondary education, university degree) corresponding to the highest level achieved. Physical activity was assessed by the IPAQ (International Physical Activity Questionnaire) questionnaire and expressed as weekly energy expenditure expressed in metabolic equivalent task minutes per week (METs min/week). Smokers were defined as current smokers, whereas non-smokers included never smokers and former smokers. The participants were also interviewed about their history of CVD and if they were diagnosed and treated for hypertension, diabetes and dyslipidemia by a medical doctor.

### Dietary pattern derivation

Dietary patterns were obtained from daily intakes of 45 food-groups using PCA and RRR. Both methods extract uncorrelated linear combinations of intakes of food groups called dietary patterns. In each technique, the extracted DP score for each participant was calculated as a sum of the food intake variables weighted by the loadings generated by the method. Factor loadings represent the correlations of each food group with the DP score. Thus, factor loadings with positive values indicate that the corresponding food groups are positively associated with the DP, whereas negative values indicate an inverse association. A large factor loading value indicates a greater contribution of that food group to the DP. Food items with absolute loadings values superior to 0.2 were considered as contributing highly to the pattern [20]. The generated dietary patterns were named according to the loadings of the foods on the factor. For every DP, a score was computed for each individual indicating the degree to which the participant’s diet conforms to the pattern. PCA aims to construct uncorrelated linear combinations of food intakes that explain as much variation in food intake as possible. In contrast, RRR aims to construct uncorrelated linear combinations of food intakes that explain as much variation in response variables as possible (25). While PCA identifies a combination of foods frequently eaten together, RRR pattern represents the combination of foods that better explains variation in a set of response variables. In this study, CVRF of interest (BMI, WHR, SBP, DBP, FPG, HbA1c, TG, LDL-C and HDL-C) were considered as response variables.

### Statistical analyses

Total and gender-specific samples were described according to socio-demographic and lifestyle characteristics as well as CVRF. Prevalence values of CVRF were calculated as described previously. Categorical variables were expressed as frequencies (n) and percentages (%) whereas continuous variables were expressed as median and interquartile range (IQR). Differences across gender were tested by a Chi-square test for categorical variables and a Wilcoxon rank sum test for continuous ones. Dietary patterns were derived using PCA and RRR. In order to keep the comparison between the two methods simple, only the first two DP of each method were retained. Given one PCA and RRR pattern, a concordant simplified dietary pattern was calculated by only considering food groups associated with both PCA and RRR pattern. Considered food groups were those with loadings of same sign and absolute loading values superior to 0.2 in PCA and RRR. Used loadings values for those food-groups in the computation of the “concordant” pattern were either -1 or 1 depending on the sign of the loading in PCA and RRR [21]. Food groups with absolute loading superior to 0.2 in one method and inferior to 0.1 in the other method were considered not concordant. Firstly, DP were described according to the loadings of each food group intakes. Then, DP were presented according to nutrient intakes, socio-demographic and lifestyle factors. Finally, separate multivariable models were used to assess linear relationships between DP (independent variables) and CVRF (dependent variables). Because DP were related to socio-demographic and lifestyle characteristics, the first model was adjusted for gender, age, educational level, smoking status and the level of physical activity. The second model was further adjusted for the medication use for the corresponding CVRF. Men and women were combined for analysis, as gender-specific DP were very similar. Moreover models tested including an interaction term indicated that there was no gender interaction. P-values < 0.05 were considered as significant. All analyses were conducted with SAS version 9.4 (SAS Institute Inc., Cary, NC, USA).

## Results

### Study population characteristics

Table 1 describes characteristics of the studied population. The sample consisted of 2298 individuals (1158 men and 1140 women) with a median age of 46.3 [34.8; 57.6] years. A significantly higher percentage of women had primary education. Concerning lifestyle characteristics, men were more likely to smoke but they were more physically active than women. With regards to CVRF, men showed higher values of BMI, WHR, SBP, DBP, FPG, TG, LDL-C and lower values of HDL-C. Consequently, a greater percentage of men suffered from, obesity/overweight, hypertension, diabetes and dyslipidemia. However, the prevalence of diabetes across gender was not significant (P = 0.17).

**Table 1:** Sociodemographic characteristics, lifestyle characteristics and cardiovascular risk factors of participants by gender (n = 2298).

	Total (n = 2298)	Men (n = 1158)	Women (n = 1140)	p-value
Sociodemographic characteristics				
Age (years)	46.3 [34.8;57.6]	45.8 [34.4;57.6]	46.9 [35.2;57.7]	0.57
Educational level				0.02
Primary, n (%)	331 (14.5%)	146 (12.7%)	185 (16.4%)	
Secondary, n (%)	1127 (49.5%)	593 (51.6%)	534 (47.4%)	
Tertiary, n (%)	818 (35.9%)	410 (35.7%)	408 (36.2%)	
Region				0.41
Luxembourg, n (%)	1071 (46.6%)	534 (46.1%)	537 (47.1%)	
Wallonia, n (%)	750 (32.6%)	392 (33.8%)	358 (31.4%)	
Lorraine, n (%)	477 (20.8%)	232 (20%)	245 (21.5%)	
Lifestyle characteristics				
Smokers, n (%)	484 (21.1%)	271 (23.4%)	213 (18.7%)	0.055
Energy expenditure per week (MET)	2490 [1059;5040]	2643 [1147;5397]	2358 [1017;4878]	0.026
Cardiovascular risk factors				
Obesity/Overweight				
Obesity/overweight (%)	1175 (51.1%)	725 (62.6%)	450 (39.5%)	<0.0001
BMI (kg/m2)	25.1 [22.5;28.6]	26.3 [23.8;29.1]	24 [24.5;27.6]	<0.0001
WHR	0.88 [0.81;0.94]	0.93 [0.88;0.98]	0.81 [0.77;0.88]	<0.0001
Hypertension				
Hypertension (%)	761 (33.1%)	483 (41.7%)	278 (24.4%)	<0.0001
Use of anti-hypertensive medication, n (%)	327 (14.2%)	192 (16.6%)	135 (11.8%)	0.001
SBP (mmHg)	126 [116;137.5]	131.5 [122;141.5]	120 [111;131.5]	<0.0001
DBP (mmHg)	78 [71.5;86]	80.5 [73.5;88]	76 [69.5;83.5]	<0.0001
Diabetes				
Diabetes (%)	100 (4.4%)	57 (4.95%)	43 (3.8%)	0.17
Use of anti diabetic medication, n (%)	72 (3.1%)	39 (3.38%)	33 (2.9%)	0.52
FPG (mmol/L)	90.6 [84.4;97.9]	92.8 [87.6;100.3]	87.6[82.3;94.1]	<0.0001
Hba1c, n (%),	5.5 [5.3;5.7]	5.5 [5.3;5.7]	5.5 [5.3;5.7]	0.06
Dyslipidemia				
Dyslipidemia (%)	1718 (74.9%)	904 (78.2%)	814 (71.6%)	0.0003
Use of serum lipid-lowering medication, n (%)	223 (9.7%)	138 (11.9%)	85 (7.5%)	0.0003
TC (mg/dl)	202 [177;230]	201 [176;229]	203 [178;232]	0.19
TG (mg/dl)	89 [64;126]	100 [72;148]	79.9 [59;111]	<0.0001
LDL-C (mg/dl)	125 [102;149]	129 [107;152]	120 [98;146]	<0.0001
HDL-C (mg/dl)	59 [49;71]	52.6 [44;62]	66 [57;77]	<0.0001

Categorical variables are expressed as n and % and continuous variables are expressed as median and IQR [P25; P75]. BMI: Body mass index; WHR: Waist to hip ratio; SBP: Systolic blood pressure; DBP: Diastolic blood pressure; FPG: Fasting plasma glucose; HbA1c: glycated haemoglobin; TC: Total cholesterol; TG: Triglycerides; LDL-C: Plasma low density cholesterol; HDL-C: plasma high density cholesterol.

### Dietary patterns

Percentage of variance explained and loadings of food groups on DP are presented in Table 2. The two PCA-patterns accounted for 10.9% (6.4% and 4.5%, respectively) of the total variance in food groups and 6.8% (4.7% and 2.1%, respectively) of the variance in CVRF (Table 2). The first PCA-pattern was labelled “Prudent” as it was characterized by high intakes of fruits, oleaginous and dried fruits, soups, vegetables, pulses, fish, low-fat dairy products, olive oil, water and tea and low intakes of fried foods, meat, ready meal, minarine and margarine, fresh cream and dressing, salty biscuits, soft drinks and beer. The second PCA-pattern was named “Animal protein and alcohol” since it was positively associated with vegetables, all kinds of meat and fish and alcohol beverages and negatively associated with sugar and sweet, low-fat dairy products, pastries and cereals.

**Table 2:** Factor loadings and explained variation of dietary patterns (n = 2298).

	Dietary pattern			
Food group	Prudent		Animal protein and alcohol	
	PCA	RRR	PCA	RRR
White bread		-0.11		**0.21**
Brown bread	0.16	**0.25**		
Cereals	0.14		**-0.24**	**-0.43**
Pastries	-0.15		**-0.29**	
Potatoes				**0.40**
Rice/pasta		**-0.32**	0.11	
Fried foods	**-0.35**	**-0.27**	0.13	0.15
Fruits	**0.50**	**0.22**		
Olieaginous fruits	**0.22**	0.19		
Dried fruits	**0.30**	**0.22**		
Soups	**0.25**	0.16		
Vegetables	**0.55**	**0.3**	**0.25**	
Pulses	**0.21**		0.20	0.13
Preserved vegetables				
Lean meat	**-0.24**	**-0.24**	**0.39**	**0.20**
Fatty meat	**-0.45**	**-0.25**	**0.3**	
Offals	0.14		**0.26**	**0.26**
Unprocessed smoked meat	-0.12		**0.26**	0.18
Processed meat	**-0.34**	**-0.28**	**0.28**	
Fish	**0.42**	0.16	**0.44**	0.19
Smoked fish	0.16		**0.41**	
Shellfish and Mussels			**0.46**	0.14
Eggs			0.10	
Ready-meals	**-0.38**	**-0.46**	0.10	
Low-fat dairy products	0.15	0.17	**-0.23**	-0.13
High-fat dairy products	**0.25**			
Soya products	0.17		-0.11	-0.16
Butter and Lower fat butter		**0.34**		
Minarine and margarine	**-0.25**			
Olive oil	**0.35**	**0.26**		-0.11
Fat rich omega6	**0.30**	**0.34**		
Fat rich in omega3	0.15	0.14		0.14
Fresh cream and dressing	**-0.36**	-0.17		-0.18
Light Fresh cream and dressing				-0.11
Sugar and sweets	-0.11	0.14	**-0.50**	**-0.31**
Salty biscuits	**-0.32**	-0.13		-0.19
Water	**0.24**	-0.16		
Coffee		**0.32**		**0.31**
Fruit or vegetables juice		-0.15	-0.13	-0.17
Soft drinks	**-0.43**	**-0.35**	-0.17	**-0.26**
Diet soft drinks	-0.20	-0.18		
Beer	**-0.23**	**-0.26**	**0.28**	**0.35**
Wine		0.14	**0.34**	**0.46**
Aperitifs and spirits		-0.18	**0.28**	**0.21**
Tea	**0.34**	**0.23**	-0.12	-0.19
Explained variation in food groups, %	6.4	4.1	4.5	3.4
Explained variation in CVRF, %	4.7	1.2	2.1	6.2

For simplicity, absolute loadings <0.1 were left out and high absolute loadings (>0.2) in bold.

Regarding RRR results, the first pattern explained 3.4% of the variation in food groups and 6.2% of the variation in CVRF whereas the second pattern accounted for 4.1% of the variation in food groups and 1.2% of the variation in CVRF. The first RRR-pattern was also named “Animal protein and alcohol” since it was also characterized by increased consumption of alcohol, offal, lean meat and decreased intakes of cereals, sugar and sweets. Those concordant food groups were used to construct the “Animal protein and alcohol” concordant-pattern. Correlation between scores of “Animal protein and alcohol” PCA- and RRR-patterns was equal to 0.57 and correlation between their corresponding factor loadings was equal to 0.63. Additionally, the RRR-pattern was also positively associated with intakes of white bread, potatoes and coffee. The second RRR-pattern was also named “Prudent” since it was also negatively associated with fried foods, lean meat, fatty meat, processed meat, ready meal, soft drinks and beers and positively associated with fruits, oleaginous and dried fruits, vegetables, olive oil and tea. Those concordant food groups were used to construct the “Prudent” concordant-pattern. Correlation between scores of “Prudent” PCA- and RRR-patterns was equal to 0.61 and correlation of corresponding factor loadings was equal to 0.73. In addition, the RRR-pattern was also negatively correlated with rice/pasta and positively correlated with butter and coffee.

### Association of DP with nutrient intakes

Table 3 displays the correlations between DP and energy-adjusted nutrient intakes. All “Prudent” patterns were positively associated with intakes of vitamins and minerals, total fiber and plant protein. Moreover, they were negatively associated with energy from alcohol, animal protein, added sugar and dietary cholesterol. “Prudent” PCA-pattern was positively correlated to energy from carbohydrate and negatively correlated to energy from lipid whereas “Prudent” RRR-pattern was positively associated with energy from lipid and inversely related to energy from carbohydrate. “Animal protein and alcohol” patterns were inversely associated with intakes of vitamins and minerals except iron and vitamin D. However, adherence to those patterns were related to higher energy from alcohol, animal protein and dietary cholesterol and lower energy from carbohydrates, added sugar.

**Table 3:** Correlation coefficients between dietary patterns and energy-adjusted nutrients (n = 2298).

Nutrients and vitamins		Dietary pattern					
		Prudent			Animal protein and alcohol		
		PCA	RRR	Concordant	PCA	RRR	Concordant
Nutrients	Alcohol	-0.12*	-0.11*	-0.18*	0.47*	0.53*	0.71*
	Carbohydrates	0.1*	-0.13*	0.17*	-0.52*	-0.15*	-0.39*
	Total fiber	0.65*	0.28*	0.58*	-0.03	-0.001	-0.21*
	Plant protein	0.34*	0.1*	0.3*	-0.07*	0.09*	-0.14*
	Animal protein	-0.08*	-0.15*	-0.3*	0.57*	0.2*	0.39*
	Added sugar	-0.34*	-0.14*	-0.2*	-0.53*	-0.46*	-0.42*
	Fat	-0.06*	0.29*	0.03	0.05*	-0.25*	-0.14*
	Cholesterol	-0.22*	-0.06*	-0.31*	0.39*	0.09*	0.28*
Vitamins	β-carotene	0.45*	0.23*	0.41*	0.11*	-0.08*	-0.06*
	Vitamin C	0.44*	0.11*	0.43*	-0.03	-0.07*	-0.08*
	Vitamin E	0.21*	0.26*	0.26*	0.03	-0.04*	-0.11*
	Iron	0.29*	0.004	0.09*	0.41*	0.08*	0.11*
	Vitamin D	0.27*	0.12*	0.1*	0.44*	0.09*	0.07*
	Calcium	0.45*	0.15*	0.33*	-0.11*	-0.14*	-0.17*

*P<0.05.

### Association of DP with sociodemographic and lifestyle characteristics

Univariate standardized β-coefficients for DP according to sociodemographic and lifestyle factors are shown in Table 4. Older subjects, women, non-smokers and individuals living in Lorraine showed higher adherence to prudent patterns. In addition, “Prudent” PCA- and concordant-patterns were also more adopted by subjects engaged in physical activity. In contrast, “Animal protein and alcohol” patterns were more likely to be adopted by men and smokers. Similarly to prudent patterns, older individuals were more likely to score higher on those patterns. Concerning the region, individuals living in Lorraine (resp in Wallonia) were more (resp less) likely to adopt a “Prudent” pattern. Compared to individuals living in Luxembourg, people in Wallonia and Lorraine were more likely to adopt an “Animal protein and alcohol” pattern.

**Table 4:** Associations between dietary patterns and sociodemographic and lifestyle characteristics (n = 2298).

	Dietary pattern					
	Prudent			Animal protein and alcohol		
Socio demographics and lifestyle characteristics	PCA	RRR	Concordant	PCA	RRR	Concordant
Age	0.34 ± 0.02**	0.39± 0.02**	0.32 ± 0.02**	0.09± 0.02**	0.39 ± 0.02**	0.14 ± 0.02 **
Gender						
Men	-0.49 ± 0.04**	-0.59 ± 0.05**	-3.22±0.21 **	0.34 ± 0.04**	0.48 ± 0.04**	1.55 ± 0.13 **
Women	Reference			Reference		
Educational level						
Primary	-0.05 ± 0.06	-0.07 ± 0.07	-0.39 ± 0.35	-0.08 ± 0.07	0.28 ± 0.07**	0.21 ± 0.21
Secondary	-0.14 ± 0.05**	-0.04 ± 0.05	-0.66 ± 0.24 *	-0.09 ± 0.05	0.21 ± 0.05**	0.17 ± 0.15
Tertiary	Reference			Reference		
Region						
Lorraine	0.41 ± 0.05**	0.59 ± 0.06**	2.16 ± 0.28 **	0.06 ± 0.06	0.31 ± 0.06**	0.97 ± 0.17 **
Wallonia	-0.39 ± 0.05**	-0.05 ± 0.05	-1.64 ± 0.24 **	0.09 ± 0.05*	-0.003 ± 0.05	0.18 ± 0.15
Luxembourg	Reference			Reference		
Smoking status						
Smokers	-0.53 ± 0.05**	-0.31 ± 0.06**	-2.71 ± 0.26 **	0.26 ± 0.05**	0.18 ± 0.05**	1.11 ± 0.16 **
No smokers	Reference			Reference		
Energy expenditure per week	0.10 ± 0.02**	0.02 ± 0.02	0.08 ± 0.02 **	-0.04 ± 0.02	0.02 ± 0.02	-0.014 ± 0.02

Data are standardized β-coefficients ± standard error.

** p <0.001.

* p <0.05

### Association of DP with CVRF

Multivariate-adjusted standardized β-coefficients for CVRF according to DP are shown in Table 5. All “prudent patterns” were inversely related to obesity (BMI and WHR) and hypertension (SBP and DBP). However, only “Prudent” RRR-pattern was associated with lipid biomarkers. Higher score on this pattern was associated with higher HDL and lower TG levels. In contrast, “Animal protein and alcohol” patterns were all positively associated with blood pressure and obesity measures as well as FPG and TG. “Animal protein and alcohol” RRR-pattern was the only DP being associated significantly with higher LDL level. No significant interactions between DP and treatment were observed and further adjustment on treatment did not change the estimates.

**Table 5:** Associations between dietary patterns and CVRF (n = 2298).

		Dietary pattern					
		Prudent			Animal protein and alcohol		
CVRF	Model	PCA	RRR	Concordant	PCA	RRR	Concordant
BMI	M1	-0.09**	-0.16**	-0.13**	0.17**	0.18**	0.11**
WHR	M1	-0.04*	-0.08**	-0.06**	0.08**	0.12**	0.11**
SBP	M1	-0.09**	-0.11**	-0.11**	0.08**	0.11**	0.08**
	M2	-0.08**	-0.09**	-0.1**	0.07**	0.1**	0.07**
DBP	M1	-0.07**	-0.06*	-0.09**	0.1**	0.12**	0.08**
	M2	-0.06**	-0.05*	-0.08**	0.09**	0.11**	0.08**
FPG	M1	-0.02	-0.02	-0.03	0.07**	0.09**	0.07*
	M2	-0.01	-0.01	-0.01	0.04**	0.07**	0.05*
Hba1c	M1	0.01	-0.01	0	0.03	0.05*	0.02
	M2	0.02	0.01	0.01	0.01	0.03	0.01
TG	M1	-0.01	-0.11**	-0.04	0.08**	0.09**	0.1**
	M2	-0.01	-0.11**	-0.04	0.08**	0.08**	0.09**
LDL-C	M1	-0.02	0.04	-0.02	0.03	0.05*	0.02
	M2	-0.01	0.04	-0.02	0.03	0.05*	0.02
HDL-C	M1	-0.02	0.1**	-0.01	0.03	0.02	0.06*
	M2	-0.01	0.1**	-0.01	0.03	0.02	0.06*

Data are standardized β-coefficients. All Standard error were equal to 0.2. M1: adjusted on gender, age, educational level, smoking status, physical activity. M2: M1 + treatment for the studied CVRF.

** p <0.001.

* p <0.05.

BMI: Body mass index; WHR: Waist to hip ratio; SBP: Systolic blood pressure; DBP: Diastolic blood pressure; FPG: Fasting plasma glucose; HbA1c: glycated hemoglobin; TC: Total cholesterol; TG: Triglycerides; LDL-C: Plasma low density cholesterol; HDL-C: plasma high density cholesterol.

## Discussion

The objectives of this study were first to compare behavior- and CVRF-related DP derived by PCA and RRR and then to explore their associations with CVRF in individuals living in the Greater Region. Behavior- and CVRF-related patterns were very similar, indicating that patterns were both predictive of CVRF and behaviorally meaningful. “Prudent” patterns were associated with a decreased cardiovascular risk whereas “Animal protein, alcohol” patterns were associated with an increased cardiovascular risk.

### Comparison of PCA- and RRR-derived patterns

Taking into account that PCA derives DP reflecting dietary behavior in a population and that RRR derives DP possibly associated with the disease of interest, the dietary methods applied in the same population do not necessarily lead to same patterns. However, in the present study, both PCA- and RRR-derived patterns seemed to be very similar.

Using the PCA-patterns as being the most behaviorally meaningful patterns, we found that the RRR-patterns also reflect eating behaviors of the population. Indeed, both “Prudent” PCA- and RRR-patterns were very similar (r = 0.61). Comparing concordant food groups between the two dietary pattern methods, they were both characterized by high intakes of fruits, oleaginous and dried fruits, vegetables, olive oil, fats rich in omega 6 and tea and low intakes of fried foods, lean and fatty meat, processed meat, ready meal, soft drink and beer. Concerning non-concordant food groups, “Prudent” RRR-pattern was also positively correlated with coffee and butter and negatively correlated with rice/pasta, whereas “Prudent” PCA-pattern was associated with intakes of water, pulses and high-fat dairy products and low intakes of minarine and margarine. “Animal protein and alcohol” PCA- and RRR-pattern were also positively correlated (r = 0.57). Comparing concordant food groups between PCA and RRR, they were both associated with low intakes of cereals, sugar and sweets and soft drinks and high intakes of fried foods, meat, offal, beer, wine and aperitifs and spirits. Concerning non-concordant food groups, “Animal protein and alcohol” RRR-pattern was also positively associated with white bread, potatoes and coffee whereas the corresponding PCA-pattern was positively associated with vegetables, fatty meat, processed meat, smoked fish and low intakes of pastries. Those similarities and correlations between PCA- and RRR-patterns show that, in our study, RRR-patterns were also behaviorally meaningful. Technically, if response variables can be explained by dietary habits of the studied population, then PCA and RRR-patterns are likely to be similar. Our findings are in line with other studies which have used CVRF as response variables and also found similar PCA- and RRR-patterns [8,9]. Likewise, comparable patterns were found when biomarkers [22,23] were chosen as response variables. Nevertheless, different patterns were found when macro-nutrients were used as response variables [24,25].

Comparing associations between CVRF and DP, we found that although the relationship with CVRF was always stronger for RRR-patterns, PCA-patterns were also associated with CVRF. All “Prudent” patterns were inversely associated with obesity and hypertension measures. However, standardized β-coefficients corresponding to the RRR-pattern were 117% and 98% higher for BMI and WHR respectively and 52% and 38% higher for SBP and DBP (compared to the PCA-pattern). In contrast to the “Prudent” PCA-pattern, the “Prudent” RRR-pattern was significantly associated with HDL cholesterol and triglycerides levels. With regards to the “Animal protein and alcohol” RRR-pattern, besides being the sole pattern associated with LDL cholesterol levels, it was more strongly associated with BP, BMI, WHR, FPG and TG than the associated PCA-pattern. Consistent with our findings, other studies also found that RRR-derived patterns were more strongly associated with response variables and other outcomes of interest than PCA-derived patterns [22,24,25].

Since RRR uses response variables to determine combinations of foods that explain a maximum amount of response variation, RRR-derived patterns are by definition associated with response variables. Response variables can be either disease outcomes of interest or variables (i.e. nutrients or biomarkers) assumed to be on the causal pathway from diet to outcomes of interest. In the first case, RRR-derived patterns will be by definition associated with the outcomes. However, in the second case, the association of RRR-patterns with outcomes depends on the strength of the relationships between response variables and outcomes. In our study, since we were interested to find dietary patterns correlated with CVRF, we chose intentionally our outcomes of interest, CVRF, as response variables. Similar to other studies that have used their outcomes measures as response variables [8,9,26], RRR-patterns were more strongly associated with outcomes than PCA patterns. Nevertheless, studies which use response variables assumed to be on the pathway between diet and the outcomes also showed stronger associations for RRR-patterns [7,27]. To our knowledge, only one study has showed stronger associations for PCA-patterns [23].

### Comparison with others studies

In accordance with other studies, we identified prudent patterns characterized by high consumption of brown bread, vegetables, fruits, oleaginous and dried fruits, fish, vegetable oil and tea and low consumption of fried foods, fatty and processed meat, ready meals, fresh cream and dressings, salty biscuits, soft drinks and alcohol [28]. The particularity of the “Prudent” RRR-pattern identified in this study was its positive association with butter intake. In addition to the prudent pattern, the majority of studies also derived a “Western” pattern characterized by a high intake of meat and processed meats, high fat content foods, refined grains, sweets and sugar, high-sugar drinks and alcoholic beverages [29,30]. Although “Animal protein and alcohol” patterns shared common elements with the Western pattern (high intakes of alcoholic beverages and meat), they were also characterized by low intakes of foods characterizing a western pattern (sugar and sweet and soft drinks). In addition, as in prudent patterns they were positively correlated with fish intakes. Likewise, other studies also failed to derive a Western pattern and similarly to us derived patterns rich in fish and meat, vegetables, alcohol and low intakes of sugar and sweet and soft drinks [31].

We found that dietary patterns were associated with socio-demographic and lifestyle characteristics. In line with other studies, women and individuals with a higher level of education seemed to adhere more to prudent patterns [20,32]. Although we found age directly correlated to prudent patterns, it has been found directly and inversely associated with prudent patterns [20,33,34]. Concerning lifestyle factors, as published previously, non-smokers were more likely to score higher on prudent patterns [35,36]. These findings are in accordance with the results of previously published studies, in which a healthy DP was found to be associated with an overall healthy lifestyle [20].

Associations found between dietary patterns and CVRF were in the expected direction. In line with the extant literature, we showed that prudent patterns were associated with lower BMI, WHR, SBP, DBP, TG [37] and higher HDL cholesterol levels [38] [39,40]. In contrast with other studies, negative associations between prudent patterns and fasting plasma glucose were not observed [41]. Regarding the “Animal protein and alcohol” patterns, it was positively associated with BMI, WHR, SBP, DBP, FPG, TG and LDL-C. Newby et al which also derived a “protein and alcohol” pattern found a direct association with BMI [31] and HDL [37]. Likewise, a meat [42] pattern and a “meat and potatoes” [6] pattern have also been positively related to BMI.

Unexpectedly, vegetables and pulses intakes were positively associated with “Animal protein and alcohol” PCA-patterns. Other authors [43] have also reported that vegetables and pulses intakes were positively correlated with patterns associated with increased risk [8], and this might be due to the fact that vegetables may be associated with intakes of meats and fats rather than being associated with increased cardiovascular risk [8]. Moreover, in our study, vegetables and pulses intakes were positively associated with the behavior-related-patterns and not with the CVRF-related-patterns which may strengthened this hypothesis. Likewise, fish intake was also positively correlated with “Animal protein and alcohol” which was associated with increased risk. This can also be explained by the fact that fish are also often consumed by protein-eater and is likely to be consumed fried in this pattern rather than being associated with increased risk. Butter intake was also positively correlated to the “Prudent” RRR-pattern. It could be argued that although butter is considered as a harmful component, it could also be part of healthy dietary habits. Since in the Greater Region, butter is often used during breakfast, its consumption could be associated with having breakfast rather than being associated with decreased risk. Unfortunately, in absence of data on breakfast habits and cooking techniques, this work could not determine whether butter was used during breakfast or for preparing meals. However, since the “Prudent” RRR-pattern was additionally positively associated with consumption of olive oil, fats rich in omega 6 and 3, it is conjectured that within this prudent pattern, butter is rather used during breakfast and vegetable oil for cooking.

Other studies assessing associations between dietary patterns and CVD excluded individuals who had a history of cardiovascular diseases and also those diagnosed with diabetes, hypertension or hyperlipidemia [9,44]. Indeed, the reason for excluding them is that those individuals with existing risk factors may have changed their diet and/or use medication for their condition. The application of those exclusion criteria would lead to a biased sample consisted mainly of healthy individuals who did not develop cardiovascular risk factors. As a result, in this study, it was decided to exclude only individuals declaring to be on diet and who had a history of cardiovascular disease. Relating to the use of medications, as in similar studies [37], it was controlled by adjustment of multivariable model on treatment.

### Robustness of the results

To assess the robustness of our findings, we conducted several sensitivity analyses. To address concerns that the results are not reproducible in other data sets, we randomly split the sample in half and ran separate analyses in the 2 subsamples. We also examined factor solutions separately for men and women. DP were also constructed after exclusion of participants diagnosed with CVRF. All dietary patterns were very similar than those computed on the entire sample. The effect of alcohol intakes on the results was also assessed. Firstly, adjustment of multivariable models on alcohol intakes did not change the strength of associations. Secondly, we found that new dietary patterns, derived without using the three food groups related to alcohol, were similar to the original patterns (correlations between corresponding patterns were all superior to 0.9 and differences of corresponding factor loadings were all comprised between -0.1 and +0.1). Finally, in order to assess if a specific cardiovascular risk factor was associated with a particular dietary pattern, RRR were performed again with a different set of response variables corresponding to different risk factors. SBP and DBP were used as response variables to define hypertension; LDL, HDL and TG levels were used to represent dyslipidemia; FPG and HbA1c for diabetes and BMI and WHR for obesity. According to the percentage of explained variation, only the first patterns of each analysis were retained. All patterns were similar with correlation coefficients between the original RRR-pattern and the dyslipidemia-related pattern equal to 0.74, 0.83 for the diabetes-related pattern, 0.86 for the hypertension-related pattern and 0.96 for the obesity-relate pattern.

### Strengths and limitations

The main strength of this study was the application and the comparison of two methods to derive dietary patterns, PCA and RRR. Moreover, we included participants with diagnosed CVRF. Adjusting food intakes on energy intake with the residual method was also necessary [14,45]. Indeed, on the opposite of absolute intakes, energy-adjusted intakes describe diet composition and control for the possible confounding effect of total energy intake [14]. Finally, it is the first study describing dietary patterns in the Greater region which has the advantage to represent a diversity of diets with participants from three different countries.

The main limitation was related to the cross-sectional design of the study. However, to limit the possibility of reverse causality, individuals on diet and with a history of cardiovascular event were excluded. Secondly, the nutritional data were derived from a self-reported FFQ, which is prone to measurement errors. However, notable correlations with biomarkers of nutritional status [11], strength of observed relationships between DP and CVRF and the use of energy-adjusted food intakes suggest a low-impact of measurement error on the results [46]. Other limitations of this study were the subjective decisions made at many levels of a dietary pattern analysis that can influence the final interpretation. The pooling of different food items into specific food groups, the way how the input variables should be quantified, whether the input variables should be adjusted for total energy intake, the number of factors to extract, the choice of a factor loadings cut-off level, the labelling of the factors are all likely to influence the final interpretation. However, the robustness of the derived DP and their relationships with CVRF have been shown by the consistency of our results. In addition, since dietary patterns may be part of a larger pattern of healthy or unhealthy behaviors, they may simply be a surrogate measure for other variables [44]. However, even after we statistically controlled for many potentially confounding variables, dietary patterns alone were still significantly associated with CVRF. Finally, although the public health message that comes from a dietary pattern approach may seem easy, their utility as a tool for public health recommendations and in helping direct public policy remains questionable. In fact, it is difficult to say if it is the full combination of food components which triggers the beneficial or adverse health effect of the pattern or only certain components.

### Conclusion

In sum, we showed that PCA and RRR methods complement each other and their application together allows to assess the similitude between real-world behavior and CVRF-related patterns. In our study, the high level of similarity between obtained PCA and RRR-patterns showed however, that these patterns were both related with CVRF and representative of eating patterns of the population. By considering food groups associated with both PCA and RRR-derived patterns, we can hypothesize that in the Greater region, a prudent pattern characterized by high intakes of fruits, oleaginous and dried fruits, vegetables, olive oil, fats rich in omega 6 and tea and low intakes of fried foods, lean and fatty meat, processed meat, ready meal, soft drink and beer reflect a dietary habit in the population and is associated with lower CVRF. By contrast, a pattern characterized by high intakes of fried foods, meat, offal, beer, wine and aperitifs and spirits and low intakes of cereals, sugar and sweets and soft drinks was associated with higher CVRF.

## Supporting Information

S1 Table (DOCX)

## Abbreviations

FFQ: Food Frequency Questionnaire
CVRF: Cardiovascular risk factors
PCA: Principal component analysis
RRR: Reduced rank regression
TC: Plasma total cholesterol
LDL: Plasma low density cholesterol
HDL: plasma high density cholesterol
BMI: Body mass index
SBP: Systolic blood pressure
DBP: Diastolic blood pressure
FPG: Fasting plasma glucose
HbA1c: glycated hemoglobin
WHR: Waist to hip ratio
TG: Triglycerides
