# Correction: Association of Empirically Derived Dietary Patterns with Cardiovascular Risk Factors: A Comparison of PCA and RRR Methods

**DOI:** 10.1371/journal.pone.0163837

**Published:** 2016-09-22

**Authors:** The PLOS ONE Staff

The fourth author’s name is spelled incorrectly. The correct name is: Leila Sissani. The sixth author’s name is spelled incorrectly. The correct name is: Saverio Stranges. The correct citation is: Sauvageot N, Leite S, Alkerwi A, Sissani L, Zannad F, Stranges S, et al. (2016) Association of Empirically Derived Dietary Patterns with Cardiovascular Risk Factors: A Comparison of PCA and RRR Methods. PLoS ONE 11(8): e0161298. doi:10.1371/journal.pone.0161298. The publisher apologizes for the error.
