# Correction: Correction: Association of Empirically Derived Dietary Patterns with Cardiovascular Risk Factors: A Comparison of PCA and RRR Methods

**DOI:** 10.1371/journal.pone.0171468

**Published:** 2017-01-30

**Authors:** The PLOS ONE Staff

## Notice of Republication

This Correction was republished on October 14, 2016, to correct errors in the citation that had been incorrectly excluded from the Correction. The publisher apologizes for the error. Please download this article again to view the correct version. The originally published, uncorrected article and the republished, corrected article are provided here for reference.

## Supporting Information

S1 File Originally published, uncorrected article. (PDF)

S2 File Republished, corrected article. (PDF)
