# Supplementary material for: Association of Empirically Derived Dietary Patterns with Cardiovascular Risk Factors: A Comparison of PCA and RRR Methods
Source: PLoS One. 2016 Aug 22;11(8):e0161298. doi: 10.1371/journal.pone.0161298 (PMC4993423; doi:10.1371/journal.pone.0161298)
Supplement: S1 Table — (DOCX) [file pone.0161298.s001.docx]

# **Table: Components of food groups included in dietary pattern**

| Foods or food groups | Food items |
| --- | --- |
| White bread | Sliced white bread; White bread; Crispbreads |
| Brown bread | Sliced brown bread; Brown bread |
| Cereals | Non-sugary cereals; Sugary cereals; Muesli |
| Pastries | Pastries; Flaky or shortcrust pastries |
| Potatoes | Potatoes |
| Rice/pasta | Rice/Pasta |
| Fried foods | Fried foods |
| Fruits | Citrus fruits; Berries; Kiwi; Banana; Pear, apple, pineapple, watermelon, melon, lychee; Plum, grape, cherry, nectarine, apricot; Fruits in syrup; Compotes |
| Oleaginous fruits | Salted dried fruits; Walnuts; Other non-salted dried fruits |
| Dried fruits | Figs /dates/prune/dried apricots |
| Soups | Vegetable soup without meat; Vegetable soup with meat |
| Vegetables | Peppers; Carrots; Salads; Green leafy vegetables; All cabbage; Other vegetables; Avocado; Olives |
| Pulses | Pulses |
| Preserved vegetables | Preserved vegetables |
| Lean meat | Pork (loin, tenderloin), beef (tournedos, steak, mince), veal escalope, chicken breast, turkey, beef(entrecote, mince), veal (breast, mince), chop, poultry mince, whole chicken, rabbit, grilled meats, lamb chop |
| Fatty meat | Breast of lamb, brest of beef, mixed mince, meatballs, lard, sausages, spare ribs, "cervelas" sausage, meatloaf, spirlingue, mutton |
| Offals | Offal |
| Unprocessed smoked meat | Unprocessed smoked cold meats |
| Processed meat | Processed meats |
| Fish | White fish;Fish; canned fish |
| Smoked fish | Smoked fish |
| Shellfish and Mussels | Shellfish; Mussels |
| Eggs | Eggs |
| Ready-meals | Prepared dishes with cod; Dressed sauerkraut; Judd mat Gardebounen; Stuffed pasta; Paella; Pizza; Quiches with meat or fish; Riesling Pâté; Fast food hamburger; Loempia, nem; Cheese croquettes, cheese crepes; Prepared fish |
| Low-fat dairy products | Plain semi-skimmed milk; Plain skimmed milk; Buttermilk; Low fat yoghurts; Low-fat yoghurts, Yakult, actimel, soft white cheeses; Lower fat cheeses |
| High-fat dairy products | Plain whole milk; Flavoured milk; Natural whole yoghurts; Sweetened or fruit whole yoghurts; Soft rind cheeses; Hard and blue cheeses; Dairy desserts; |
| Soya products | Plain soya milk; Flavoured soya milk. |
| Butter and Lower fat butter | Butter and lower fat butter |
| Minarine and margarine | Minarine and margarine |
| Olive oil | Olive oil |
| Fat rich in omega6 | Oil and margarine rich in omega6 |
| Fat rich in omega3 | Oil and margarine rich in omega3 |
| fresh cream and dressing | fresh cream and dressing |
| Light fresh cream and dressing | Light fresh cream and dressing |
| Sugar and sweets | Jam, honey; Chocolate spread; Chocolate; Peanuts butter; Dry biscuits; Chocolate snacks; Flavoured ice creams; Ice cream products; Jellified desserts; Brown or white sugar; Sweets; Cocoa |
| Salty biscuits | Aperitif biscuits/Crisps |
| Water | Water |
| Coffee | Coffee |
| Fruit or vegetables juice | Fruit juice; Vegetable juice. |
| Soft drinks | Soft drinks |
| Diet soft drinks | Diet soft drinks and alcohol-free beers |
| Beer | Light beers pils; Strong beers |
| Wine | Wines |
| Aperitifs and spirits | Aperitifs; Spirits |
| Tea | Tea; Infusions |
