# Supplementary material for: Correction: Correction: Association of Empirically Derived Dietary Patterns with Cardiovascular Risk Factors: A Comparison of PCA and RRR Methods
Source: PLoS One. 2017 Jan 30;12(1):e0171468. doi: 10.1371/journal.pone.0171468 (PMC5279807; doi:10.1371/journal.pone.0171468)
Supplement: S1 File — (PDF) [file pone.0171468.s001.PDF]

PLOS | ONE

CORRECTION

Correction: Association of Empirically Derived Dietary Patterns with Cardiovascular Risk Factors: A Comparison of PCA and RRR Methods

Nicolas Sauvageot, Sonia Leite, Ala'a Alkerwi, Leila Sisanni, Faiez Zannad, Saverio Stranges, Anne-Françoise Donneau, Adelin Albert, Michèle Guillaume

The sixth author's name is spelled incorrectly. The correct name is: Saverio Stranges. The correct citation is: Sauvageot N, Leite S, Alkerwi A, Sisanni L, Zannad F, Stranges S, et al. (2016) Association of Empirically Derived Dietary Patterns with Cardiovascular Risk Factors: A Comparison of PCA and RRR Methods. PLoS ONE 11(8): e0161298. doi:10.1371/journal.pone.0161298

Reference

1. Sauvageot N, Leite S, Alkerwi A, Sisanni L, Zannad F, Saverio S, et al. (2016) Association of Empirically Derived Dietary Patterns with Cardiovascular Risk Factors: A Comparison of PCA and RRR Methods. PLoS ONE 11(8): e0161298. doi:10.1371/journal.pone.0161298 PMID: 27548287

CrossMark
click for updates

OPEN ACCESS

Citation: Sauvageot N, Leite S, Alkerwi A, Sisanni L, Zannad F, Stranges S, et al. (2016) Correction: Association of Empirically Derived Dietary Patterns with Cardiovascular Risk Factors: A Comparison of PCA and RRR Methods. PLoS ONE 11(9): e0163837. doi:10.1371/journal.pone.0163837

Published: September 22, 2016

Copyright: © 2016 Sauvageot et al. This is an open access article distributed under the terms of the Creative Commons Attribution License, which permits unrestricted use, distribution, and reproduction in any medium, provided the original author and source are credited.
